# Looking for the X Factor in Bacterial Pathogenesis: Association of *orfX*-*p47* Gene Clusters with Toxin Genes in Clostridial and Non-Clostridial Bacterial Species

**DOI:** 10.3390/toxins12010019

**Published:** 2019-12-31

**Authors:** Maria B. Nowakowska, François P. Douillard, Miia Lindström

**Affiliations:** Department of Food Hygiene and Environmental Health, Faculty of Veterinary Medicine, University of Helsinki, 00014 Helsinki, Finland; maria.nowakowska@helsinki.fi (M.B.N.); francois.douillard@helsinki.fi (F.P.D.)

**Keywords:** botulinum neurotoxin, neurotoxin gene cluster, *orfX*, *p47*, neurotoxin associated proteins

## Abstract

The botulinum neurotoxin (BoNT) has been extensively researched over the years in regard to its structure, mode of action, and applications. Nevertheless, the biological roles of four proteins encoded from a number of BoNT gene clusters, i.e., OrfX1-3 and P47, are unknown. Here, we investigated the diversity of *orfX-p47* gene clusters using in silico analytical tools. We show that the *orfX-p47* cluster was not only present in the genomes of BoNT-producing bacteria but also in a substantially wider range of bacterial species across the bacterial phylogenetic tree. Remarkably, the *orfX-p47* cluster was consistently located in proximity to genes coding for various toxins, suggesting that OrfX1-3 and P47 may have a conserved function related to toxinogenesis and/or pathogenesis, regardless of the toxin produced by the bacterium. Our work also led to the identification of a putative novel BoNT-like toxin gene cluster in a *Bacillus* isolate. This gene cluster shares striking similarities to the BoNT cluster, encoding a *bont/ntnh*-like gene and *orfX-p47*, but also differs from it markedly, displaying additional genes putatively encoding the components of a polymorphic ABC toxin complex. These findings provide novel insights into the biological roles of OrfX1, OrfX2, OrfX3, and P47 in toxinogenesis and pathogenesis of BoNT-producing and non-producing bacteria.

## 1. Introduction

Botulinum neurotoxin (BoNT) is mostly produced by the Gram-positive spore-forming anaerobic bacterium *Clostridium botulinum*. BoNT causes botulism which is a rare but deadly disease affecting both humans and animals [1]. The most well-known form of the disease is food-borne botulism, resulting from the consumption of preformed BoNT present in inappropriately preserved food products [2,3]. Upon ingestion and absorption into the body, BoNT, a zinc-dependent metalloprotease, blocks neurotransmission through the cleavage of key proteins within the cholinergic nerve terminals, causing flaccid paralysis [4]. BoNT is encoded from neurotoxin gene cluster(s) (NGC) which can be located either in the chromosome or within mobile genetic elements, i.e., plasmids or bacteriophages, and acquired through horizontal gene transfer [5]. The NGC typically encodes several components of the active progenitor toxin complex (PTC), as well as regulatory elements related to toxinogenesis [6].

The gene organization and content of NGCs differ substantially between botulinum neurotoxin producing strains. Two major NGC types are recognized based on the type of genes localized in direct vicinity of the BoNT-encoding gene (*bont*): the hemagglutinin (*ha*) type and the *orfX-p47*-type NGCs. Both cluster types, besides carrying *bont*, contain five to six additional genes which encode known or putative neurotoxin-associated proteins (NAPs) and an alternative sigma factor, BotR. Regardless of the NGC type, BoNT is exclusively encoded with the non-toxic non-hemagglutinin protein (NTNH) which interlocks with BoNT to form a minimally functional PTC (M-PTC). Formation of the M-PTC shields the naturally fragile BoNT and protects it from degradation in the harsh conditions of the gastrointestinal tract [7]. The *ha*-type NGCs encode three hemagglutinins (HA-17, HA-33, and HA-70) which link with the M-PTC to form a large PTC (L-PTC) [8,9]. The L-PTC facilitates the transport of BoNT through the intestinal epithelial barrier enabling the toxin to enter circulation [10,11,12]. In contrast, the *orfX-p47* type NGC does not harbor hemagglutinin genes. The *ha17*, *ha33*, and *ha70* are replaced with genes named *orfX1*, *orfX2*, and *orfX3*. Despite the lack of structural homology between OrfX1-3 and the HA proteins [13], an identical operon structure and location next to the *bont*-*ntnh* operon supports a hypothesis that the proteins encoded by *orfX1*, *orfX2*, and *orfX3* might also hold a role in PTC formation or in BoNT pathogenesis. However, the roles of OrfX1, OrfX2 (PDB ID: 6EKV), and OrfX3 proteins remain to be elucidated. Along with the *orfX1-3* genes, the *orfX-p47* type NGCs exclusively harbor a gene (*p47*) encoding a 47-kDa product (PDB ID: 5WIX, 6EKT) of an unknown function.

OrfX1, OrfX2, OrfX3, and P47 remain poorly characterized and only few studies investigated their structures and biochemical properties. Biochemical analysis showed that OrfX1, OrfX2, and P47 attach to lipids in vitro [13,14]. The lipid-binding properties of OrfX2 and P47 were attributed to tubular lipid-binding (TULIP) domains so far encountered only in eukaryotic proteins [13]. Occasionally, the OrfX1, OrfX2, OrfX3, and P47 proteins were detected in association with BoNT immune-extracted from *C. botulinum* cultures or with commercially available purified BoNT complexes [15]. However, their presence varied significantly between samples, which suggests that specific conditions may be required to capture a putative L-PTC containing OrfX1, OrfX2, OrfX3, and/or P47. Besides the putative roles of OrfX1, OrfX2, OrfX3, and/or P47 as structural components of the L-PTC, roles in regulation of NGC expression have been proposed [16] but not experimentally supported [17]. A better understanding of P47, OrfX1, OrfX2, and OrfX3 would shed light on the mode of action of BoNTs encoded from the *orfX-p47*-type NGCs.

Until recently, BoNT production was merely associated with clostridial species including *C. botulinum* and some strains of *Clostridium argentinense*, *Clostridium baratii*, and *Clostridium butyricum* [6,18,19]. Rapid development of next-generation sequencing (NGS) technologies and bioinformatics tools allowed affordable genome sequencing and functional genomic analyses of clinical and environmental isolates, and resulted in in silico discovery of novel BoNT types, not only in *Clostridiales* but also in phylogenetically distant bacterial species [20,21,22,23]. Along these lines, the present study, based on bioinformatic mining of bacterial genome databases, led to the identification of genes putatively encoding OrfX1, OrfX2, OrfX3, and P47 in bacterial species belonging to *Alphaproteobacteria*, *Bacilli*, *Betaproteobacteria*, *Cytophagia*, and *Gammaproteobacteria*. Diverse and novel *orfX1*, *orfX2*, *orfX3*, and *p47* gene arrangements as well as the presence of a truncated form of *orfX2* (tentatively called *orfX-T*), previously unseen in *Clostridiales*, were reported in these genomes. Strikingly, these genes were consistently neighboring genes encoding non-BoNT toxins, including the crystal toxin (Cry) or vegetative insecticidal proteins (VIP). This suggests that the biological function of OrfX1, OrfX2, OrfX3, and P47 relates to toxinogenesis and/or pathogenesis, and is not BoNT-specific. Finally, we identified an *orfX-p47*-containing toxin gene cluster harboring a *bont/ntnh*-like toxin gene in a *Bacillus* isolate. This cluster shares similarities with *C. botulinum* NGC but also possesses marked differences: besides having three *orfX* genes (*orfX3* and two *orfX-T*) and a *p47*, it contains a solitary truncated *bont/ntnh*-like gene lacking the canonically accompanying *ntnh/bont* counter partner. Instead, this cluster is juxtaposed by two open reading frames which putatively encode components of a tripartite toxin complex. This finding suggests that the truncated BoNT/NTNH-like protein detected in a *Bacillus* isolate may display a mode of action and host specificity different from canonical BoNTs. Together, the present work reveals new insights into the biological roles of OrfX1, OrfX2, OrfX3, and P47 in bacterial toxinogenesis and pathogenesis.

## 2. Results and Discussion

### 2.1. Phylogenetic Distribution of orfX1, orfX2, orfX3, and p47 in Bacteria

**Figure 1:**
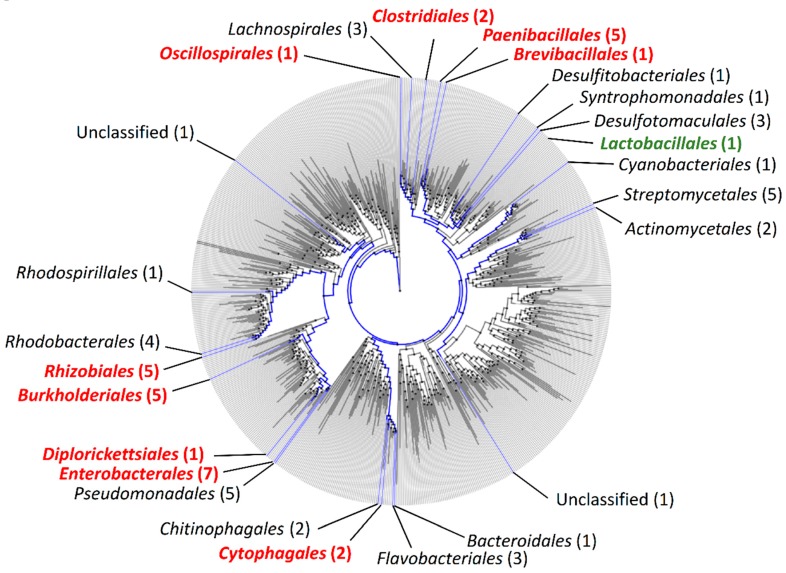
Phylogenetic distribution of the PF06597 domain (*Clostridium* P47) among bacterial species based on Annotree. Blue branches correspond to species carrying the PF06597 domain (*Clostridium* P47 superfamily). Strains/species belonging to the orders shown in red were further analyzed in the present study. The order of *Lactobacillales* is highlighted in green as it was not detected in Annotree, although this order has one known *E. faecium* isolate harboring a neurotoxin gene cluster with the *orfX-p47* genes.

The BoNT gene cluster frequently harbors *orfX1*, *orfX2*, *orfX3*, and *p47* in BoNT-producing *Clostridia*. While the *ha* operon has been exclusively linked to BoNT gene clusters of *Clostridia*, the *orfX1-3* operon and *p47* have also been found in BoNT gene clusters of phylogenetically more distant species, such as *Enterococcus faecium* [21]. We hypothesized that *orfX1-3* and *p47* may be found in a wider range of bacterial species. As OrfX2, OrfX3, and P47 belong to the *Clostridium* P47 superfamily (Pfam ID: PF06597), we searched for the PF06597 domain using the online web tool Annotree (Figure 1). Bacterial species belonging to 23 different orders, such as *Bacteroidales*, *Rhizobiales*, or *Streptomycetales*, were identified as encoding *Clostridium* P47 superfamily proteins. As Annotree includes only representative genomes and thus omits intraspecies genomic diversity, it is possible that the analysis does not fully reflect the true phylogenetic distribution of the superfamily PF06597. Therefore, it is likely that proteins belonging to the *Clostridium* P47 superfamily are present even in a larger number of taxonomic orders or families. This is in contrast with the phylogenetic distribution of BoNT and BoNT-like proteins, suggesting that the biological function of *Clostridium* P47 superfamily proteins is not exclusively associated with BoNT. Further evidence supporting this hypothesis is discussed below.

**Figure 2:**
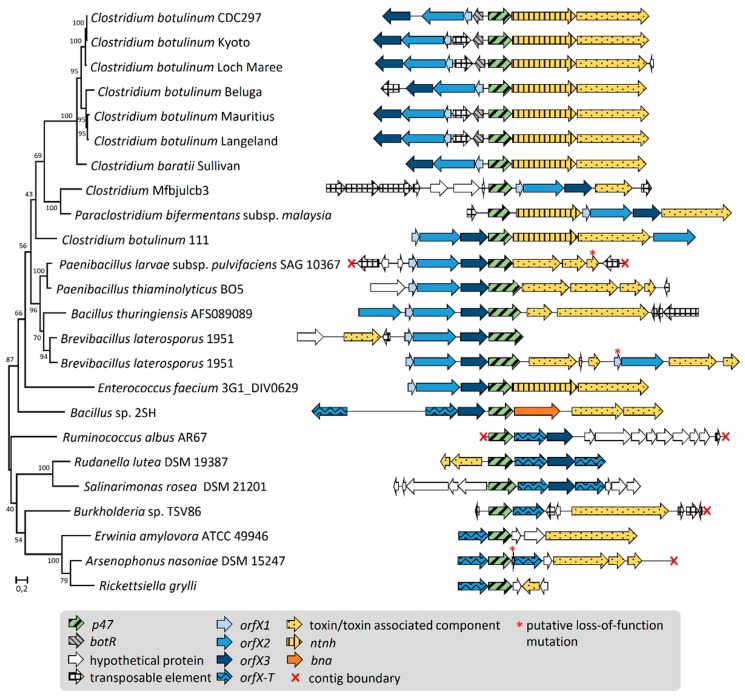
Gene arrangement of *orfX-p47* clusters. Each chromosomal region was ordered based on the maximum likelihood phylogenetic tree of P47. Values indicated on the tree branches are bootstrapping values (250 iterations).

We also searched for putative homologs of *Clostridium* P47, OrfX1, OrfX2, and OrfX3 using protein BLAST in the ‘non-redundant protein sequence (nr)’ database within and beyond the order *Clostridiales*, to identify putative novel *orfX-p47* gene clusters. In line with the Annotree search, OrfX1, OrfX2, OrfX3, and P47 homologs were detected in bacterial species belonging to various taxonomic orders (Table 1, Figure 1, and Supplementary Tables S1–S5). In most genomes analyzed, the genes encoding OrfX1-3 and P47 homologs were arranged in clusters. While the copy number and presence of the different *orfX1-3* genes varied between the identified clusters, each cluster always displayed one copy of *p47* (Figure 2). Additionally, most of the detected novel *orfX-p47* clusters resided genes encoding components of toxins other than BoNT. In few cases, we detected orphan genes for proteins harboring the *Clostridium* P47 superfamily. Of note, we did perform a similar search for *Clostridium* hemagglutinin homologs beyond the *Clostridiales* order but failed to detect any significant hits, suggesting that the *ha* gene clusters are conserved to *Clostridiales*, as opposed to *orfX-p47* genes.

### 2.2. Gene Organization and Arrangement of orfX1, orfX2, orfX3, and p47

To perceive the diversity of OrfX1, OrfX2, OrfX3, and P47 encoding loci, we compared the gene arrangement of the OrfX1-3 and P47 protein homologs retrieved from the genomes of thirteen bacterial strains (Table 1). These strains originated from various environmental samples, i.e., air, insects, sediment, soil, and water samples. Most of these bacterial species are either invertebrate pathogens (*Brevibacillus laterosporus*, *Bacillus thuringiensis*, *Rickettsiella grylli*, *Paenibacillus larvae*) or plant pathogens (*Erwinia amylovora*). All the analyzed *orfX-p47* clusters harbored one copy of *p47*. We, therefore, constructed a maximum likelihood tree of all P47 proteins (Figure 2) and juxtaposed the corresponding *orfX-p47* gene clusters according to the tree. Phylogenetically related bacterial species harbored clusters with similar gene arrangement and order. In most Gram-positive bacteria, the overall arrangement of *orfX1-3* and *p47* was unidirectional and well-conserved with the following order: *orfX1*, *orfX2*, *orfX3*, and *p47* (Figure 2). In contrast, in *C. botulinum* strains, the orientation of *p47* is opposite to the *orfX1*, *orfX2*, and *orfX3* and occasionally flanked by a regulator gene or a mobile element. The *orfX-p47* gene cluster in *C. botulinum* strain 111 encoding BoNT/X markedly differs from the ones in other *C. botulinum* strains, as previously reported, and its gene arrangement rather resembles the *orfX-p47* clusters found in phylogenetically more distant species, such as *Bacilli*, *Brevibacilli*, and *Paenibacilli* where *p47* is localized downstream the *orfX1-3* operon. Remarkably, there is a clear dichotomy in the architecture of the *orfX-p47* gene clusters between Gram-negative and Gram-positive species: *orfX1* was present within the *orfX-p47* gene cluster of Gram-positive species but was absent in clusters from all analyzed Gram-negative bacteria. Considering the recently demonstrated lipid binding ability of OrfX1 in vitro, it is tempting to speculate that the biological function of OrfX1 could relate to membranes in the Gram-positive cell envelope. Gram-negative species, in turn, lacked *orfX1*, and carried one or two copies of truncated *orfX2*, tentatively called *orfX-T*, and occasionally harbored *orfX3*.

### 2.3. P47, OrfX1, OrfX2, and OrfX3 Have a Common Origin

**Figure 3:**
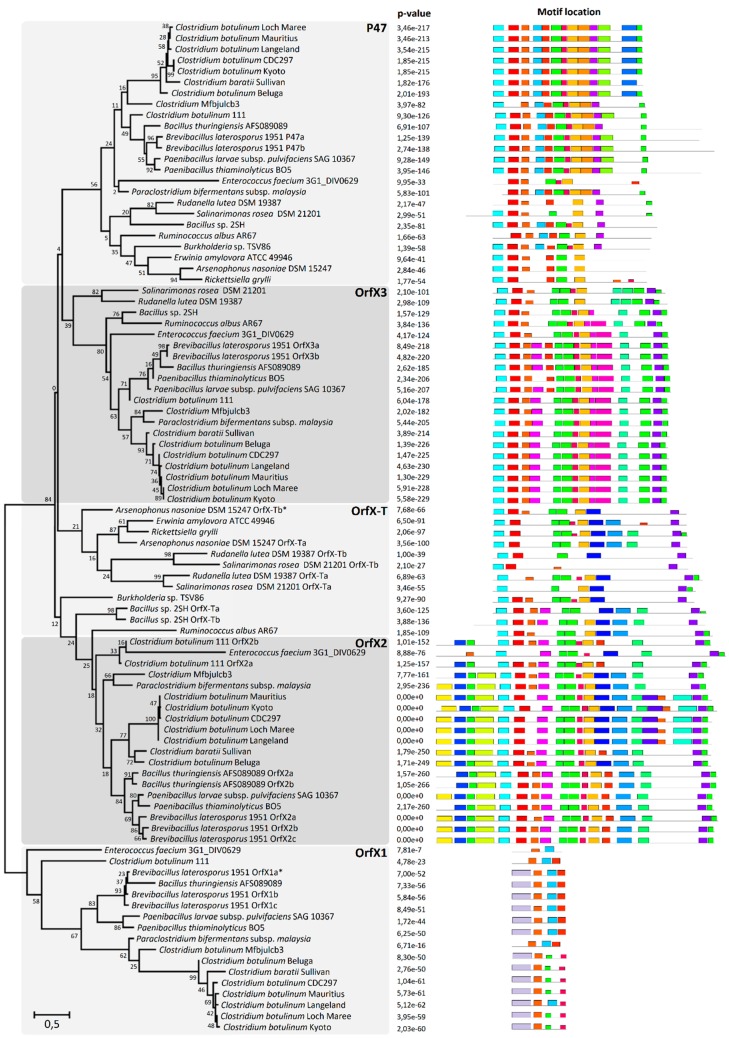
Maximum likelihood phylogenetic tree of P47, OrfX1, OrfX2, OrfX3, and OrfX-T. Values indicated on the tree branches are bootstrapping values (250 iterations). The results retrieved from MEME analysis of all protein sequences were juxtaposed to the corresponding strains (*p*-values and motif locations). The color-coded blocks correspond to different amino acid motifs detected within the analyzed protein sequences and serve to visualize the similarities between the proteins.

Since P47, OrfX2, and OrfX3 all belong to the *Clostridium* P47 superfamily, we further examined the domain conservation and the evolutionary relationship between these proteins found in phylogenetically distinct bacterial species. We constructed a maximum likelihood tree of all OrfX1-3 and P47 proteins and collated it with the corresponding MEME (Multiple EM for Motif Elicitation) analysis which detected any shared and recurring amino acid motifs or patterns in a color-coded graphical form. All analyzed protein sequences grouped into five main clades, namely clade P47, and clades OrfX1, OrfX2, OrfX3, and OrfX-T (Figure 3). OrfX1 diverged early from the other clades, retaining only a few motifs that were also present in the other clades. Remarkably, all the analyzed Gram-negative and some Gram-positive species, e.g., *Bacillus* sp. 2SH and *Ruminoccus albus*, were devoid of OrfX1 and OrfX2 but instead harbored OrfX-T, a truncated form of OrfX2. OrfX-T shares a number of unique motifs with OrfX2, but lacks the N-terminal domain of OrfX2 which appears to be associated with the presence of OrfX1. The fact that the clusters harboring *orfX-T* always lacked *orfX1* and intact *orfX2* may suggest that OrfX-T alone takes over the biological functions of both OrfX1 and OrfX2. The MEME analysis showed that P47, OrfX2, and OrfX3 share several conserved motifs inside the core domain, suggesting that the three proteins originate from a common ancestor (Figure 3). There is also a high degree of conservation among three consecutive motifs located in the N-terminal domains of P47 and OrfX3 and in the core domain of OrfX2, and among other three motifs found within the core domains of each of the three proteins. P47 and OrfX3 share similar N-terminal and core domains but possess different motifs within their C-terminal domains. While the C-terminal domain of P47 appears to be unique, the one in OrfX3 shares similarities with the C-terminal domain of OrfX2.

Intriguingly, the novel *orfX-p47* clusters identified in *B. laterosporus* (VIP toxin producer), *B. thuringiensis* (Cry toxin producer) and *P. thiaminolyticus* (VIP toxin producer) encoded an extended P47 with an additional domain fused to its C-terminus. The additional domain detected in P47 was predicted to contain a ricin-type beta-trefoil lectin-like domain (Pfam ID: PF14200), involved in carbohydrate binding [32] and found for instance in HA-33 [33]. Furthermore, one of the two *orfX-p47* clusters harbored by *B. laterosporus* carrying a gene for Cry toxin, encoded P47 fused to a domain identified as a fungal immunomodulatory protein Fve domain (Pfam ID: PF09259) that may bind cell-surface carbohydrates [34]. The identification of these two distinct types of cell-binding domains fused to P47 suggests a putative role for P47 as a cell-binding anchor (lectin-like domain or fungal immunomodulatory protein Fve domain) that could dictate target specificity of the associated toxins produced by these isolates.

### 2.4. Association of orfX1, orfX2, orfX3, and p47 with Toxin Genes

Most of the *orfX-p47* clusters were adjacent to genes encoding various well-studied insecticidal toxins like Cry toxin (classified as delta-endotoxin) or VIP (belonging to the group of binary toxins) (Table 2). A significant number of putative toxin genes associated with *orfX-p47* clusters encoded proteins containing different types of motifs or domains correlated with the toxic properties. Among these, we have identified proteins harboring rearrangement hotspot (RHS) repeats present in a wide range of insecticidal toxins [35,36], ribosome inactivating protein domains [37], or bacterial immunoglobulin-like domains, the latter shown to be associated with intestinal colonization [38] and recently detected within the BoNT-like toxin encoded by *Weissella oryzae* [39]. Of note, these toxins share several common features, including oral infection route, proteolytic activation, and an ability to form pores in the target host cells [40,41,42,43].

Interestingly, literature describing Cry and VIP toxins, or bacterial genomes encoding them, has not recognized the presence of OrfX or P47-encoding genes in the close neighborhood of the toxin genes. To the best of our knowledge, only one report shows the presence of *orfX-p47* cluster in a non-BoNT-encoding genome, however this finding has not been further investigated [44]. Our BLAST analysis showed that *cry* and *vip* genes are seldom accompanied by *orfX-p47* clusters. Moreover, Cry toxin is biologically effective alone, for example when used in pest control, thus OrfXs and P47 are likely not essential in Cry pathogenesis. We assume that in the absence of OrfX1-3 and P47, potency of the toxins may be lower, albeit sufficient to kill the host. Alternatively, other as-yet unidentified accessory proteins may assist in toxinogenesis. It will be important to experimentally verify if the association of Cry and VIP toxins with OrfX and P47 proteins impacts their potency. Our data mining also revealed several assumingly orphan *orfX-p47* clusters neighboring genes encoding hypothetical proteins. It is not clear whether these genes co-operate with *orfX1-3* and *p47*, whether they encode toxic components, and whether they are coincidentally co-localized with the *orfX-p47* clusters as a result of a phage or mobile element activity.

### 2.5. Identification of BoNT/NTNH-Like Protein in Bacillus sp. 2SH

**Figure 4:**
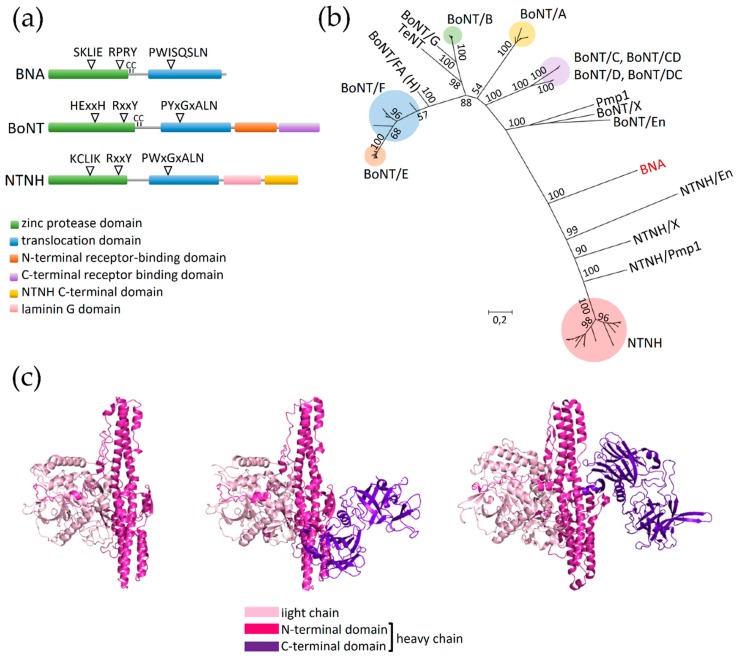
Protein sequence analysis of BNA. (**a**) Domain structure of BNA compared to the canonical domain structure of BoNT and NTNH. The NCBI conserved domain detection tool was used to predict protein domains within BNA, BoNT, and NTNH protein sequences. (**b**) Maximum likelihood phylogenetic tree of BoNT, NTNH, TeNT, and BNA. Values indicated on the tree branches are bootstrapping values (250 iterations). (**c**) Structural modeling of BNA based on I-TASSER prediction (left) compared to the structures of NTNH type D (middle) and BoNT type B (right).

Bioinformatic analysis led to the discovery of an *orfX-p47* cluster-associated gene putatively encoding a protein with similarities to BoNT and NTNH. We termed this putative protein as BoNT/NTNH-like A component (BNA) due to its close similarity to BoNT and NTNH and to its predicted function as component A of an ABC-type toxin complex (discussed below). This peculiar cluster was identified within the unclosed genome of a *Bacillus* sp. isolate 2SH recovered from alpine fresh water in Trento, Italy. The predicted 825 amino acid long sequence of BNA protein was compared with an extensive protein dataset consisting of various types/subtypes of BoNT. Preliminary identification of conserved protein domains revealed that BNA contains the clostridial neurotoxin zinc protease domain (Pfam ID: PF01742) characteristic for both BoNT and NTNH, and a clostridial neurotoxin translocation domain (Pfam ID: PF07952) which is identified in BoNT, NTNH and the tetanus neurotoxin (TeNT). Interestingly, the predicted BNA polypeptide chain lacked the C-terminal heavy chain domain present in both BoNT and NTNH (Figure 4a). The amino acid sequence alignment suggested that BNA carries several conserved motifs present in BoNT and NTNH. They include an active site-stabilizing motif RxxY [45] and a translocation motif PWISQSLN, which in BoNT is conserved as PYxGxALN and in NTNH as PWxGxALN [46]. Interestingly, BNA demonstrates the presence of two cysteines (C376 and C386) located between the BNA zinc protease and translocation domain. Homologous residues found in BoNT allow the formation of an inter-chain disulfide bond, which upon reduction enables the toxin to translocate across the target-cell membranes [47,48] (Figure 4a). The conserved active site zinc-coordinating HExxH motif present in zinc metalloproteases, including BoNT, is absent in BNA. Instead, BNA contains the amino acid sequence SKLIE, undetected in any of the known BoNTs. The SKLIE sequence does not contain two histidine residues necessary for chelating catalytic zinc ions in botulinum and tetanus zinc metalloproteases [49]. However, it remains to be experimentally determined whether the SKLIE motif is functional and in which conditions, and what would constitute the molecular target of BNA. The findings suggest that BNA cannot bind to the target cell receptors typically recognized by BoNT and most likely cannot incorporate a zinc residue that is indispensable in BoNT for proteolytic cleavage of its target proteins; however it potentially could form a translocation channel.

Within the maximum likelihood phylogenetic tree, BoNT and NTNH clustered distinctly as previously reported [50]. Although the BNA amino acid sequence clustered with the NTNH clade, its early branching places it between BoNT and NTNH (Figure 4b). This, together with the fact that the BNA gene (*bna*) does not reside with an *ntnh*-like gene, may suggest that BNA represents the common ancestor of BoNT and NTNH before their likely emergence through gene duplication [51]. On the other hand, the long phylogenetic distance between BNA and BoNT or NTNH could suggest that *bna* constitutes a pseudogenized form of *bont* or *ntnh* and is no longer functional. Nevertheless, we have not identified any premature stop codon or frameshift mutations within the *bna* sequence, which opposes the pseudogene hypothesis and supports functionality.

The putative BNA protein also shows striking structural resemblance to BoNT and NTNH. Its 3D structure predicted using the Phyre2 tool matched with the crystal structure of BoNT/B (PDB ID: 1S0B) indicating that BNA is likely highly similar to BoNT/B and, by extension, to other BoNTs. Another 3D structure-generating software, I-TASSER, modeled the structure of BNA utilizing NTNH/D (PDB ID: 3VUOA) as a template. The latter BNA model is the one presented and analyzed here (Figure 4c). This 3D model confirms the lack of C-terminal domain of the BNA heavy chain. However, the remaining light chain and N-terminal heavy chain domains share structural identity with BoNT and NTNH. Unfortunately, the degree of sequence conservation did not allow a model with a maximum confidence, therefore, a crystal structure of BNA would further validate in silico generated structural models.

The *bna* gene is lacking the canonical *bont* or *ntnh* counterpart, which makes BNA, to our knowledge, the first described stand-alone BoNT/NTNH-like protein. Instead, *bna* is accompanied by two open reading frames encoding proteins containing RHS repeats (Figure 2, *Bacillus* sp. 2SH cluster). Their 3D models match with the crystal structure of TcdB2-TccC3 toxin subcomplex of *Photorhabdus luminescens* (PDB ID: 4O9X; 100% confidence in Phyre2). This subcomplex is involved in the formation of tripartite ABC toxin of *P. luminescens* which is a well-studied example of RHS-repeat containing polymorphic toxin targeting insect larvae [52,53]. Recent studies showed that BoNT-like proteins can exhibit insecticidal potential [23], therefore the presence of *bna* next to the genes putatively encoding anti-insect toxin components did not appear coincidental. Accordingly, we discuss below whether incorporation of BNA into an ABC toxin complex can be rationalized.

Each component of the ABC toxin complex is responsible for performing a different task, and when assembled an active multimeric structure is generated. The A protein forms a pentameric structure to make a translocation channel in the target host cells [54]. Accordingly, in the case of *Bacillus* sp. 2SH putative ABC complex, BNA could act as the A component due to the fact it encompasses a BoNT translocation domain which may form translocation pores in lipid bilayers [55]. The putative self-oligomerization step would therefore stabilize BNA, explaining why BNA lacks BoNT or NTNH-like assistant. The two other ORFs localized downstream of *bna* appear to encode the B and C components which form a capsule-like structure protecting the cytotoxic hypervariable region inside the C-domain of the C component, which can exhibit different modes of toxicity [56]. The NCBI conserved domain detection tool showed that the C-domain of the putative C protein of *Bacillus* sp. 2SH consists of bacterial SNF2 helicase related to chromatin remodeling [57]. This suggests that the putative BNA-associated ABC complex toxicity could rely on rearranging the DNA of target cells. A relevant piece of evidence supporting the hypothesis of BNA being an A component of ABC toxin complex is the demonstrated interchangeability of the A component: the B and C components co-expressed with the A component of different bacterial strains can form a toxic ABC complex with variable, A component-defined host specificity [54,58]. Accordingly, we speculate that BNA could be utilized by the *Bacillus* sp. 2SH ABC toxin system as an externally acquired gatekeeper to the neuronal cells. To our knowledge, this is the first case where a BoNT/NTNH-like protein could be utilized as a component of a non-botulinum toxin system. Further experimental investigation of this putative novel toxin complex will bring more information about its target specificity, action, and evolutionary status.

## 3. Conclusions

Here we showed that the *orfX-p47* gene cluster, so far exclusively associated with botulinum neurotoxins, is widely distributed across the bacterial phylogenetic tree, reaching far beyond the distribution of the *bont-ntnh* gene pair. The *orfX-p47* cluster showed large diversity in gene arrangement and gene content, which to great extent is parallel with the phylogenetic relationships among the bacteria harboring these gene clusters. Phylogenetic analysis of OrfX1-3 and P47 protein sequences suggested that the four proteins originate from a common ancestor and evolved through the acquisition or loss of functional domains. The Gram-negative bacteria possessing *orfX-p47* clusters harbored atypical *orfX* genes in comparison to their putative Gram-positive orthologs. Different cell envelope architecture between Gram-negative and Gram-positive bacteria and evidence of OrfX1, OrfX2, and P47 binding to lipids in vitro [13,14] may suggest a role for OrfX proteins related to the bacterial cell envelope. This hypothesis awaits further testing by studying of the localization of the OrfX proteins within the bacterial cells.

Remarkably, the *orfX-p47* clusters were consistently associated with genes encoding various types of oral insecticidal toxins, i.e., delta-endotoxins (Cry toxin), binary toxins (VIP toxin) or ABC toxins. This suggests that the biological role of OrfX1-3 and P47 is not specific to BoNT. These proteins rather play a general role in oral toxinogenesis and pathogenesis of bacteria. Although the mode of action of OrfX proteins is unknown, recent work in *Paraclostridium bifermentans* subsp. *malaysia* showed that the co-expression of OrfX proteins may increase the oral toxicity of the mosquitocidal toxin PMP1 [23]. The relatively high degree of conservation among OrfX and P47 (Figure 3 and Tables S1–S5) as opposed to the diversity of associated toxins (in terms of size, structure, mode of action) suggests that the OrfX and P47 proteins indirectly assist in toxin production, release, or trafficking. Further research on the insecticidal toxin-related OrfX and P47 proteins may lead to novel interventions in pest control.

Identification of the *orfX-p47* cluster in *Bacillus* sp. 2SH led to the discovery of a *bont/ntnh*-like toxin gene (*bna*) predicted to encode a protein with partial homology to BoNT and NTNH. This BoNT/NTNH-like A component appears unique since it is assumingly devoid of a canonical assistant protein. Instead, the *bna* gene is located upstream of two genes predicted to encode the B and C components of an ABC toxin complex [59]. We suggest that BNA constitutes the A component of an ABC toxin complex and therefore may have a role in determining host specificity of the toxin complex. To our knowledge, this is the first piece of evidence supporting incorporation of a BoNT/NTNH-like protein into a toxin complex different from the botulinum neurotoxin complex. Moreover, this is the first report of a *bont/ntnh* homolog in *Bacillus* sp. These data suggest that BoNT or NTNH proteins/homologs could be interchanged between different toxin complexes. This finding constitutes a basis for further studies on the interchangeability of BoNT domains with other toxins, and provides further evidence on the possible function of OrfX and P47 proteins in oral toxicity of BoNT and other bacterial toxins.

## 4. Materials and Methods

### 4.1. Sequence Database Mining and Conserved Domain Analysis

Amino acid sequences of OrfX1 (WP_003369622.1), OrfX2 (WP_003371659.1), OrfX3 (WP_003372464.1), and P47 (WP_003374133.1) of *C. botulinum* strain Beluga were used as the query sequence to perform a protein–protein BLAST (blastp) search [60] against the NCBI ‘non-redundant protein sequence (nr)’ database (July 26, 2019). Homolog searches were also performed by excluding *Clostridia* (taxid: 186801), in order to filter out all OrfX1, OrfX2, OrfX3, and P47 protein sequences present in BoNT-producing *Clostridia* genomes. All BLAST (blastp) analyses were performed using default settings (scoring parameters: BLOSUM62 matrix; gap costs: existence 11 and extension1; expected threshold 10, word size 6) (Supplementary Tables S1–S5). Conserved domains of protein homologs were further scanned and checked using the NCBI Conserved Domain Search using defined options (database: CDD v3.17 – 52,910 PSSMs, expected value threshold 0.01) [61,62,63,64]. Genome sequences and amino acid sequences of relevant protein homologs were retrieved from the NCBI database. OrfX1, OrfX2, OrfX3, and P47 protein sequences from *C. botulinum* (strains Beluga, Kyoto-F, Langeland, Mauritius, CDC_297, 111, Mfbjulcb3), *C. baratii* (strain Sullivan), *E. faecium* (strain 3G1_DIV0629), and *P. bifermentans* subsp. *malaysia* (strain Pbm) were included in the OrfX-P47 dataset for further comparative analysis. Search using the protein family corresponding to *Clostridium* P47 superfamily (Pfam ID: PF06597) was performed in Annotree [65]. Protein sequences from the genes located in the vicinity of the *orfX* gene cluster were further investigated for domain conservation (Pfam 32.0 search) [66], sequence homology (BLAST) [60], and structural homology (Phyre2) [67]. Phylogenetic distributions of the different bacterial genomes analyzed in this work were positioned within the tree generated by Annotree (taxonomic order level) [65]. The list of genome sequences analyzed in this study is shown in Table 1.

### 4.2. Comparative Sequence Analysis, Motif-Based Sequence Analysis, and Phylogenetic Tree Analysis

Sequence alignment based on ClustalW algorithm (gap opening penalty 10, gap extension penalty 0.1, protein weight matrix BLOSUM) [68] was computed in MEGA7 [69]. Sequence alignment was used to perform principal component analysis (PCA) in JalView v2.10.5 [70]. Maximum-likelihood phylogenetic trees were generated in MEGA7 with custom options (nearest-neighbor-interchange, Jones-Thornton model, 250 iterations) [69]. Putative motifs conserved among protein sequences were further analyzed using the MEME suite v.5.0.5 (MEME tool, classic mode, site distribution set to zero or one per sequence, number of motifs set to 30) [71].

### 4.3. Sequence Analysis of the Putative Toxin Gene Cluster in Bacillus sp. 2SH and Structural Modeling

Protein BLAST search, Pfam 32.0 search, and Phyre2 analysis of the gene (tentatively named BoNT/NTNH-like A component, BNA, WP_137842862.1) downstream *p47* in *Bacillus* sp. 2SH revealed sequence similarities with NTNH type B of *C. botulinum* (BAQ12789.1, 30.03% identity, E value 8 × 10^−104^, query cover 90%) and other NTNH homologs. The amino acid sequence of BNA was subsequently aligned with a dataset of BoNT, NTNH, and TeNT amino acid sequences retrieved from public databases. Sequence alignment was performed based on ClustalW algorithm (gap opening penalty 10, gap extension penalty 0.1, protein weight matrix BLOSUM) [68] in MEGA7 [69]. Maximum-likelihood phylogenetic trees were generated in MEGA7 as described above [69]. Initial protein homology modeling was performed using Phyre2 (intensive modeling mode) [67] and SWISS MODEL [72,73,74,75,76,77]. Structural modeling was also carried out using I-TASSER v5.1 [78,79]. BNA model and relevant structural templates were visualized using PyMOL [80].

## Figures and Tables

**Table 1:** Bacterial genomes analyzed in this study.

Bacterial Species	Strain Name	Taxonomy (Class)	Isolation Source	Accession Number	Reference
*Clostridium botulinum*	Beluga	*Clostridia*	Fermented whale flippers, Canada	NZ_ACSC01000002	Direct submission
*Clostridium botulinum*	Kyoto-F	*Clostridia*	Infant feces, Japan	CP001581	[24]
*Clostridium botulinum*	CDC_297	*Clostridia*	Liver paste, USA	CP006907	[25]
*Clostridium botulinum*	111	*Clostridia*	Infant feces, Japan	AP014696	[26]
*Clostridium botulinum*	Loch Maree	*Clostridia*	Duck liver paste, Scotland	CP000962	[24]
*Clostridium botulinum*	Mauritius	*Clostridia*	Fish, Mauritius	NZ_LFPL01000000	[18]
*Clostridium botulinum*	Langeland	*Clostridia*	Liver paste, Denmark	CP000728	Direct submission
*Clostridium botulinum*	Mfbjulcb3	*Clostridia*	Retail fish market, India	CP027780	Direct submission
*Clostridium baratii*	Sullivan	*Clostridia*	Adult human feces, USA	CP006905	[25]
*Enterococcus faecium*	3G1_DIV0629	*Bacilli*	Cow feces, USA	NGLI00000000	Direct submission
*Paraclostridium bifermentans* subsp. *malaysia*	Pbm	*Clostridia*	Swamp soil, Malaysia	CM017269	[23]
*Arsenophonus nasoniae*	DSM 15247	*Gammaproteobacteria*	Son-killer of *Nasonia vitripennis*, USA	AUCC00000000	Direct submission
*Bacillus* sp.	2SH	*Bacilli*	Alpine fresh spring, Italy	SCNA01000023	[27]
*Brevibacillus laterosporus*	1951	*Bacilli*	Forage rape seed, New Zealand	RHPK00000000	[28]
*Burkholderia* sp.	TSV86	*Betaproteobacteria*	Water, Australia	GCA_001522865	Direct submission
*Erwinia amylovora*	ATCC 49946	*Gammaproteobacteria*	Infected apple tree, USA	FN666575	[29]
*Paenibacillus larvae* subsp. *pulvifaciens*	SAG 10367	*Bacilli*	*Apis mellifera* (honeybee), Chile	NZ_CP020557	[30]
*Paenibacillus thiaminolyticus*	BO5	*Bacilli*	Soil, Russia	GCA_003591545.1	Direct submission
*Rickettsiella grylli*	-	*Gammaproteobacteria*	Pill bugs, USA	NZ_AAQJ00000000	Direct Submission
*Rudanella lutea*	DSM 19387	*Cytophagia*	Air sample, South Korea	NZ_ARPG00000000	Direct submission
*Ruminococcus albus*	AR67	*Clostridia*	Sheep rumen, New Zealand	GCA_900112155	Direct submission
*Salinarimonas rosea*	DSM 21201	*Alphaproteobacteria*	Salt mine sediment, China	NZ_AUBC00000000	Direct submission
*Bacillus thuringiensis*	AFS089089	*Bacilli*	Grainbin dust, USA	NVNL01000046	[31]

**Table 2:** Features of genes contiguous to the *orfX-p47* gene cluster. Predicted annotations are based on sequence homology (protein BLAST) and/or structural homology (Phyre2). The E-score corresponds to the identified domain.

Bacterial Strain	OrfX-assisting Gene	Protein ID	Predicted Annotation	NCBI Conserved Domain Search Output (Accession Number)	E-score
*Clostridium botulinum* Beluga	*bont/E*	EES49627.1	BoNT type E	Clostridial neurotoxin, translocation domain (cl06820)	3.07 × 10^−93^
				Clostridial neurotoxin zinc protease (cl15546)	1.10 × 10^−79^
				Clostridial neurotoxin, N-terminal receptor binding (PF07953)	9.90 × 10^−68^
				Clostridial neurotoxin, C-terminal receptor binding (cl08467)	3.90 × 10^−22^
	*ntnh/E*	EES49602.1	NTNH protein	Clostridial neurotoxin zinc protease (cl15546)	1.15 × 10^−83^
				Laminin G domain (cl22861)	1.79 × 10^−47^
				Non-toxic non-hemagglutinin C-terminal (cl07187)	2.43 × 10^−40^
*Clostridium* Mfbjulcb3	C7M59_04110	AVQ52086.1	Crystal insecticidal protein (Cry)/insecticidal delta-endotoxin	Delta-endotoxin, C-terminal domain (cd04085)	5.38 × 10^−39^
				Delta-endotoxin (cl15971)	5.32 × 10^−14^
				Delta-endotoxin, N-terminal domain (cl04339)	2.11 × 10^−13^
*Arsenophonus nasoniae* DSM 15247	NN^a^	WP_026823093.1	RHS repeat protein	RHS Repeat (PF05593)	1.19 × 10^−5^
				Uncharacterized conserved protein RhaS (COG3209)	3.84 × 10^−3^
	NN	WP_026823094.1	RHS repeat protein	-	-
	NN	WP_081700660.1	RHS repeat protein	RHS repeat-associated core domain (cl37315)	2.68 × 10^−12^
				Beta-eliminating lyase (cl18945)	2.98 × 10^−3^
*Bacillus* sp. 2SH	BNA^b^	WP_137842862.1	BoNT/NTNH-like A component (BNA)	Clostridial neurotoxin zinc protease (cl15546)	3.55 × 10^−34^
				Clostridial neurotoxin, translocation domain (cl06820)	1.14 × 10^−26^
	NN	WP_137842861.1	RHS repeat protein	-	-
	NN	WP_137842860.1	RHS repeat protein	RHS repeat-associated core domain (TIGR03696)	2.88 × 10^−26^
				Bacterial SNF2 helicase associated domain (cl07173)	1.81 × 10^−3^
*Brevibacillus laterosporus* 1951	EEL31_08340 (cluster I)	TPG68525.1	Crystal insecticidal protein (Cry)/insecticidal delta-endotoxin	Delta-endotoxin, C-terminal domain (cd04085)	1.86 × 10^−22^
				Delta-endotoxin (cl15971)	1.13 × 10^−13^
				Delta-endotoxin, N-terminal domain (cl04339)	1.03 × 10^−11^
	EEL31_17680 (cluster II)	TPG70133.1	Binary toxin/vegetative insecticidal protein (VIP1)	Clostridial binary toxin B/anthrax toxin PA domain 2 (cl38748)	2.52 × 10^−39^
				Clostridial binary toxin B/anthrax toxin PA Ca-binding domain (cl09551)	2.12 × 10^−14^
				Clostridial binary toxin B/anthrax toxin PA domain 3 (cl38749)	6.89 × 10^−12^
	EEL31_17670 (cluster II)	TPG71603.1	Vegetative insecticidal protein (VIP2)	VIP2, ADP-ribosyltransferase exoenzyme (cl00173)	4.71 × 10^−51^
				Clostridial binary toxin B/anthrax toxin PA domain 2 (cl38748)	1.60 × 10^−39^
	EEL31_17650 (cluster II)	TPG70130.1	Binary toxin/vegetative insecticidal protein (VIP1)	Clostridial binary toxin B/anthrax toxin PA Ca-binding domain (cl09551)	1.44 × 10^−14^
				Clostridial binary toxin B/anthrax toxin PA domain 3 (cl38749)	3.11 × 10^−8^
	EEL31_17645 (cluster II)	TPG70129.1	Vegetative insecticidal protein (VIP2)	VIP2, ADP-ribosyltransferase exoenzyme (cl00173)	3.92 × 10^−66^
*Burkholderia* sp. TSV86	WS68_18250	WP_059573479.1	Autotransporter protein	Outer membrane autotransporter barrel domain (cl36898)	5.76 × 10^−52^
				Autotransport protein MisL (cl36477)	1.07 × 10^−19^
				Large exoprotein involved in heme utilization or adhesion (COG3210)	2.08 × 10^−6^
				Extended signal peptide of type V secretion system (PF13018)	4.04 × 10^−5^
*Erwinia amylovora* ATCC 49946	EAM_RS01885	WP_004160289.1	RHS repeat protein	RHS repeat-associated core domain (TIGR03696)	1.12 × 10^−23^
				Uncharacterized conserved protein RhaS (COG3209)	8.37 × 10^−8^
*Paenibacillus larvae* subsp. *pulvifaciens* SAG 10367	B7C51_09885	ARF68072.1	Binary toxin/vegetative insecticidal protein (VIP1)	Clostridial binary toxin B/anthrax toxin PA domain 2 (cl38748)	4.05 × 10^−40^
				Clostridial binary toxin B/anthrax toxin PA Ca-binding domain (cl09551)	1.82 × 10^−10^
				Clostridial binary toxin B/anthrax toxin PA domain 3 (cl38749)	6.37 × 10^−7^
				PA14 domain (cl08459)	3.38 × 10^−5^
	B7C51_09880	ARF68071.1	Vegetative insecticidal protein (VIP2)	VIP2, ADP-ribosyltransferase exoenzyme (cl00173)	1.86 × 10^−8^
				Anthrax toxin lethal factor (cl08465)	2.48 × 10^−3^
	B7C51_09875	NN	Anthrax toxin lethal factor/vegetative insecticidal protein (VIP2)	VIP2, ADP-ribosyltransferase exoenzyme (PF03496)	3.59 × 10^−62^
*Paenibacillus thiaminolyticus* BO5	DQX05_07030	WP_119792154.1	Binary toxin/vegetative insecticidal protein (VIP1)	Clostridial binary toxin B/anthrax toxin PA domain 2 (cl38748)	1.14 × 10^−39^
				Clostridial binary toxin B/anthrax toxin PA Ca-binding domain (cl09551)	5.76 × 10^−15^
				Clostridial binary toxin B/anthrax toxin PA domain 3 (cl38749)	1.63 × 10^−7^
				PA14 domain (cl08459)	1.49 × 10^−4^
				Ricin-type beta-trefoil lectin domain-like (PF14200)	4.93 × 10^−3^
	DQX05_07025	WP_119792152.1	Binary toxin/vegetative insecticidal protein (VIP1)	Clostridial binary toxin B/anthrax toxin PA domain 2 (cl38748)	2.27 × 10^−41^
				Clostridial binary toxin B/anthrax toxin PA Ca-binding domain (cl09551)	1.32 × 10^−15^
				Clostridial binary toxin B/anthrax toxin PA domain 3 (cl38749)	2.02 × 10^−6^
	DQX05_07020	WP_119792150	Vegetative insecticidal protein (VIP2)	VIP2, ADP-ribosyltransferase exoenzyme (cl00173)	1.20 × 10^−6^
	DQX05_07015	WP_119792149	Anthrax toxin lethal factor/vegetative insecticidal protein (VIP2)	Anthrax toxin lethal factor (cl08465)	6.60 × 10^−4^
				VIP2, ADP-ribosyltransferase exoenzyme (cl00173)	5.05 × 10^−63^
*Rickettsiella grylli*	RICGR_0720	WP_081441678.1	Shiga toxin A-chain (rRNA N-glycosidase)	Ribosome inactivating protein (cl08249)	7.84 × 10^−20^
*Rudanella lutea* DSM 19387	NN	WP_019988042.1	BIG-5 domain containing protein	Bacterial Ig-like domain, BIG5 (PF13205)	1.62 × 10^−16^
	NN	WP_019988043.1	Low affinity iron permease	Low affinity iron permease (PF04120)	1.20 × 10^−70^
*Ruminococcus albus* AR67	SAMN02910406 _03599	WP_074963339.1	Starch-binding protein	Uncharacterized conserved protein YjdB, contains Ig-like domain (COG5492)	1.24 × 10^−10^
				Starch-binding module 26 (PF16738)	1.92 × 10^−8^
*Bacillus thuringiensis* AFS089089	CON71_23765	WP_098902378.1	Crystal insecticidal protein (Cry)	Insecticidal crystal toxin, P42 (cl05149)	5.68 × 10^−11^
	CON71_23770	WP_098902379.1	RHS repeat protein	RHS repeat-associated core domain (TIGR03696)	7.79 × 10^−25^
				Uncharacterized conserved protein RhaS (COG3209)	7.58 × 10^−7^

^a^ NN, no name/tag assigned in the deposited database. ^b^ Name assigned in the present study.

## Supplementary Materials

The following are available online at https://www.mdpi.com/2072-6651/12/1/19/s1, Table S1: BLAST analysis of P47 homologs compared to *C. botulinum* Beluga P47, Table S2: BLAST analysis of OrfX1 homologs compared to *C. botulinum* Beluga OrfX1, Table S3: BLAST analysis of OrfX2 homologs compared to *C. botulinum* Beluga OrfX2, Table S4: BLAST analysis of OrfX3 homologs compared to *C. botulinum* Beluga OrfX3, Table S5: BLAST analysis of OrfX-T homologs compared to *C. botulinum* Beluga OrfX2 and OrfX3.
