# Supplementary material for: Looking for the X Factor in Bacterial Pathogenesis: Association of orfX-p47 Gene Clusters with Toxin Genes in Clostridial and Non-Clostridial Bacterial Species
Source: Toxins (Basel). 2019 Dec 31;12(1):19. doi: 10.3390/toxins12010019 (PMC7020563; doi:10.3390/toxins12010019)
Supplement: Supplementary file 1 [file toxins-12-00019-s001.pdf]

Supplementary Materials: Looking for the X Factor in Bacterial Pathogenesis: Association of *orfX-p47* Gene Clusters with Toxin Genes in Clostridial and Non-Clostridial Bacterial Species

Maria B. Nowakowska, François P. Douillard and Miia Lindström

Table S1. BLAST analysis of P47 homologs compared to *C. botulinum* Beluga P47.

Bacterial Species	Strain	Predicted Gene Product	AA Length	Query Cover	E-score	Overall % Identity	% Identity	% Similarity	Gaps
Clostridium botulinum	Kyoto	P47	416	100%	0.0	72%	72%	83%	0%
Clostridium botulinum	Loch Maree	P47	416	100%	0.0	73%	73%	84%	0%
Clostridium botulinum	Langeland	P47	416	100%	0.0	81%	81%	91%	0%
Clostridium botulinum	Mauritius	P47	416	100%	0.0	80%	80%	89%	0%
Clostridium botulinum	CDC_297	P47	416	100%	0.0	72%	72%	83%	0%
Clostridium baratii	Sullivan	P47	416	100%	0.0	68%	68%	82%	1%
Clostridium sp.	Mfbjulcb3	P47	423	99%	4.0×10^{-45}	35%	35%	55%	6%
Clostridium botulinum	111	P47	428	99%	2.0×10^{-87}	38%	38%	58%	10%
Paraclostridium bifermentans subsp. *malaysia*	Pbm	P47 *	426	95%	4.0×10^{-81}	40%	37%	58%	4%
Paenibacillus thiaminolyticus	BO5	P47 *	577	99%	2.0×10^{-90}	36%	36%	58%	4%
Paenibacillus larvae subsp. *pulvifaciens*	SAG 10367	P47	431	99%	8.0×10^{-95}	36%	36%	58%	4%
Brevibacillus laterosporus	1951	P47b *	572	99%	1.0×10^{-94}	36%	36%	57%	4%
Brevibacillus laterosporus	1951	P47a *	614	99%	3.0×10^{-88}	36%	36%	58%	8%
Bacillus thuringiensis	AFS089089	P47 *	579	100%	2.0×10^{-81}	34%	34%	52%	8%
Enterococcus faecium	3G1_DIV0629	P47	412	66%	5.0×10^{-34}	30%	30%	50%	8%
Rudanella lutea	DSM 19387	P47	445	85%	6.0×10^{-39}	26%	26%	47%	6%
Salinarimonas rosea	DSM 21201	P47	519	81%	3.0×10^{-29}	26%	26%	44%	6%
Bacillus sp.	2SH	P47	456	99%	2.0×10^{-60}	32%	31%	50%	9%
Ruminococcus albus	AR67	P47 *	440	94%	4.0×10^{-37}	28%	28%	47%	13%
Burkholderia sp.	TSV86	P47	436	90%	2.0×10^{-38}	25%	25%	45%	10%
Erwinia amylovora	ATCC 49946	P47	425	81%	1.0×10^{-20}	22%	22%	42%	14%
Arsenophonus nasoniae	DSM 15247	P47	425	56%	1.0×10^{-14}	23%	23%	44%	10%
Rickettsiella grylli	-	P47	428	67%	9.0×10^{-21}	26%	26%	46%	11%

* % identity, % similarity and % gap correspond to the largest hit, as opposed to the overall percentage identity.

Table S2. BLAST analysis of OrfX1 homologs compared to *C. botulinum* Beluga OrfX1.

Bacterial Species	Strain	Predicted Gene Product	AA Length	Query Cover	E-score	Overall % Identity	% Similarity	Gaps
Clostridium botulinum	Kyoto	OrfX1	142	98%	2.0×10^{-65}	73%	86%	0%
Clostridium botulinum	Loch Maree	OrfX1	142	98%	1.0×10^{-64}	72%	86%	0%
Clostridium botulinum	Langeland	OrfX1	142	98%	9.0×10^{-68}	75%	87%	0%
Clostridium botulinum	Mauritius	OrfX1	142	98%	1.0×10^{-66}	73%	88%	0%
Clostridium botulinum	CDC_297	OrfX1	142	98%	5.0×10^{-74}	73%	86%	0%
Clostridium baratii	Sullivan	OrfX1	140	98%	6.0×10^{-60}	60%	80%	1%
Clostridium sp.	Mfbjulcb3	OrfX1	127	86%	3.0×10^{-31}	43%	67%	0%
Paraclostridium bifermentans subsp. *malaysia*	Pbm	OrfX1	142	97%	1.0×10^{-32}	45%	63%	0%
Paenibacillus thiaminolyticus	BO5	OrfX1	141	95%	2.0×10^{-16}	26%	58%	1%
Paenibacillus larvae subsp. *pulvifaciens*	SAG 10367	OrfX1	140	97%	1.0×10^{-19}	29%	56%	13%
Brevibacillus laterosporus	1951	OrfX1a	140	98%	2.0×10^{-19}	25%	59%	1%
Brevibacillus laterosporus	1951	OrfX1b	140	98%	5.0×10^{-19}	25%	57%	1%
Brevibacillus laterosporus	1951	OrfX1c	126	72%	1.00×10^{-9}	24%	56%	1%
Bacillus thuringiensis	AFS089089	OrfX1	140	97%	3.0×10^{-22}	29%	61%	1%
Clostridium botulinum	111	OrfX1	132	96%	2.0×10^{-9}	26%	55%	7%
Enterococcus faecium	3G1_DIV0629	OrfX1-like	134	13%	1.2×10^{-1}	40%	55%	0%

Table S3. BLAST analysis of OrfX2 homologs compared to *C. botulinum* Beluga OrfX2.

Bacterial Species	Strain	Predicted Gene Product	AA Length	Query Cover	E-score	Overall % Identity	% Similarity	Gaps
Clostridium botulinum	Kyoto	OrfX2	764	99%	0.0	51%	69%	2%
Clostridium botulinum	Loch Maree	OrfX2	750	99%	0.0	51%	69%	2%
Clostridium botulinum	Langeland	OrfX2	750	99%	0.0	51%	69%	2%
Clostridium botulinum	Mauritius	OrfX2	750	99%	0.0	51%	69%	2%
Clostridium botulinum	CDC_297	OrfX2	750	99%	0.0	51%	69%	2%
Clostridium baratii	Sullivan	OrfX2	748	99%	0.0	65%	80%	0%
Clostridium sp.	Mfbjulcb3	OrfX2	732	97%	3.0×10^{-104}	32%	48%	7%
Clostridium botulinum	111	OrfX2a	745	99%	2.0×10^{-111}	32%	50%	7%
Clostridium botulinum	111	OrfX2b	748	98%	4.0×10^{-108}	32%	50%	6%
Paraclostridium bifermentans subsp. *malaysia*	Pbm	OrfX2	740	98%	8.0×10^{-131}	34%	53%	8%
Paenibacillus thiaminolyticus	BO5	OrfX2	757	98%	2.0×10^{-125}	32%	51%	6%
Paenibacillus larvae subsp. *pulvifaciens*	SAG 10367	OrfX2	755	99%	5.0×10^{-137}	34%	51%	9%
Brevibacillus laterosporus	1951	OrfX2c	758	99%	4.0×10^{-131}	34%	52%	8%
Brevibacillus laterosporus	1951	OrfX2a	767	99%	1.0×10^{-129}	33%	52%	7%
Brevibacillus laterosporus	1951	OrfX2b	758	99%	4.0×10^{-129}	33%	52%	6%
Bacillus thuringiensis	AFS089089	OrfX2a	766	98%	1.0×10^{-128}	34%	51%	8%
Bacillus thuringiensis	AFS089089	OrfX2b	765	98%	2.0×10^{-135}	34%	52%	5%
Enterococcus faecium	3G1_DIV0629	OrfX2	787	92%	6.0×10^{-55}	26%	43%	12%

Table S4. BLAST analysis of OrfX3 homologs compared to *C. botulinum* Beluga OrfX3.

Bacterial Species	Strain	Predicted Gene Product	AA Length	Query Cover	E-score	Overall % Identity	% Similarity	Gaps
Clostridium botulinum	Kyoto	OrfX3	490	100%	0.0	77%	88%	0%
Clostridium botulinum	Loch Maree	OrfX3	490	100%	0.0	76%	88%	0%
Clostridium botulinum	Langeland	OrfX3	490	100%	0.0	77%	89%	0%
Clostridium botulinum	Mauritius	OrfX3	490	100%	0.0	77%	89%	0%
Clostridium botulinum	CDC_297	OrfX3	490	100%	0.0	77%	89%	0%
Clostridium baratii	Sullivan	OrfX3	491	100%	0.0	81%	91%	0%
Clostridium sp.	Mfbjulcb3	OrfX3	489	98%	1.0×10^{-150}	48%	63%	2%
Clostridium botulinum	111	OrfX3	488	99%	5.0×10^{-150}	47%	64%	3%
Paraclostridium bifermentans subsp. *malaysia*	Pbm	OrfX3	491	99%	2.0×10^{-168}	49%	67%	1%
Paenibacillus thiaminolyticus	BO5	OrfX3	494	98%	2.0×10^{-143}	46%	63%	1%
Paenibacillus larvae subsp. *pulvifaciens*	SAG 10367	OrfX3	494	98%	3.0×10^{-145}	46%	63%	1%
Brevibacillus laterosporus	1951	OrfX3a	489	98%	9.0×10^{-160}	48%	65%	1%
Brevibacillus laterosporus	1951	OrfX3b	489	98%	1.0×10^{-158}	48%	64%	1%
Bacillus thuringiensis	AFS089089	OrfX3	492	98%	3.0×10^{-143}	44%	64%	2%
Enterococcus faecium	3G1_DIV0629	OrfX3	489	98%	8.0×10^{-104}	38%	56%	7%
Rudanella lutea	DSM 19387	OrfX3	485	98%	2.0×10^{-63}	29%	46%	7%
Salinarimonas rosea	DSM 21201	OrfX3	479	98%	9.0×10^{-58}	28%	46%	9%
Bacillus sp.	2SH	OrfX3	488	98%	4.0×10^{-114}	39%	60%	2%
Ruminococcus albus	AR67	OrfX3	473	96%	5.0×10^{-99}	39%	56%	5%

Table S5. BLAST analysis of OrfX-T homologs compared to *C. botulinum* Beluga OrfX2 and OrfX3.

				**Compared to *C. botulinum* Beluga OrfX2**			**Compared to *C. botulinum* Beluga OrfX3**		
**Bacterial Species**	**Strain**	**Predicted Gene Product**	**AA Length**	**Query Cover**	**E-score**	**Overall % Identity**	**Query Cover**	**E-score**	**Overall % Identity**
Rudanella lutea	DSM 19387	OrfX-Ta	583	81%	1.0×10^{-29}	23%	71%	1.0×10^{-13}	23%
Rudanella lutea	DSM 19387	OrfX-Tb	556	66%	0.002	21%	65%	2.0×10^{-6}	19%
Salinarimonas rosea	DSM 21201	OrfX-Ta	564	89%	2.0×10^{-19}	22%	57%	9.0×10^{-15}	24%
Salinarimonas rosea	DSM 21201	OrfX-Tb	543	41%	3.0×10^{-6}	20%	53%	8.0×10^{-7}	20%
Bacillus sp.	2SH	OrfX-Ta	595	80%	6.0×10^{-54}	28%	54%	2.0×10^{-16}	25%
Bacillus sp.	2SH	OrfX-Tb	642	94%	1.0×10^{-55}	28%	67%	1.0×10^{-16}	26%
Ruminococcus albus	AR67	OrfX-T	601	84%	1.0×10^{-51}	27%	71%	1.0×10^{-22}	31%
Burkholderia sp.	TSV86	OrfX-T	560	84%	8.0×10^{-40}	26%	53%	7.0×10^{-26}	29%
Erwinia amylovora	ATCC 49946	OrfX-T	539	61%	5.0×10^{-24}	24%	53%	3.0×10^{-12}	23%
Arsenophonus nasoniae	DSM 15247	OrfX-Tb	540	83%	2.0×10^{-26}	23%	69%	3.0×10^{-18}	26%
Arsenophonus nasoniae	DSM 15247	OrfX-Ta	538	67%	5.0×10^{-27}	24%	58%	8.0×10^{-21}	23%
Rickettsiella grylli	-	OrfX-T	542	82%	2.0×10^{-27}	23%	87%	9.0×10^{-12}	22%
